# The Pathogenesis of *Candida* Infections in a Human Skin Model: Scanning Electron Microscope Observations

**DOI:** 10.5402/2011/150642

**Published:** 2011-09-05

**Authors:** A. Raz-Pasteur, Y. Ullmann, I. Berdicevsky

**Affiliations:** ^1^Departement of Microbiology, The Ruth and Bruce Rappaport Faculty of Medicine, Technion—Institute of Technology, P.O. Box 9649, 31096 Haifa, Israel; ^2^Infectious Diseases Unit, Rambam Health Care Campus, 31096 Haifa, Israel; ^3^Department of Plastic Surgery, Rambam Health Care Campus, 31096 Haifa, Israel

## Abstract

Cutaneous candidiasis is an opportunistic infection that arises, in most cases, from endogenous, saprophytic candidal blastospores that selectively colonize oral, gastrointestinal, vaginal, and cutaneous epithelium. *Candida albicans* has been regarded as the most common causative agent in human fungal infections. However, other *Candida* species have become a significant cause of infection. Scanning electron microscope (SEM) observations were used to analyze the capability of *C. albicans*, *C. tropicalis*, and *C. parapsilosis* to adhere to human skin model, used in this study, which was found to mimic the human skin in vivo. The skin sections were inoculated with low and high concentration of the yeasts and followed for 1 and 5 days; then they were viewed by SEM. The electron microscopy observations revealed that all three yeasts tested adhered to the skin but *C. albicans* covered the entire skin model to a higher extent than *C. tropicalis* or *C. parapsilosis*. Mucin-like material coated the blastoconidia mainly in *C. albicans*. All *Candida* species have shown characteristics resembling biofilm formation. The use of human skin sections for ex vivo evaluation of adherence of various yeasts may partially explain the predominance of *C. albicans* in cutaneous pathogenicity.

## 1. Introduction

Yeasts are unicellular fungi that typically reproduce by budding.

Candidosis is an infection caused by the yeast *Candida albicans* or other *Candida* species.

*Candida albicans* is the principal fungal infectious agent in human infection.

Superficial infections of skin and mucous membranes are the most common types of candidal infections of the skin [1].

Cutaneous candidiasis is an opportunistic infection that arises, in most cases, from endogenous, saprophytic* Candida blastospores* that selectively colonize oral, gastrointestinal, vaginal, and cutaneous epithelium [2–5].

Under various environmental conditions, *Candida blastospores* may undergo mycelial transformation, invade epithelial tissue, and evoke a complement-dependent neutrophil-mediated acute inflammatory response characterized by subcorneal pustules [6, 7].

Initial and prerequisite events in cutaneous candidiasis should, hypothetically, include colonization of epithelial surfaces with pathogenic species of *Candida*.

*Candida tropicalis*, *Candida parapsilosis*, *Candida krusei*, *Candida guilliermondii* and are less common in causing human disease.

It has been well demonstrated that specific *Candida* species, notably *C. albicans*, selectively adhere to vaginal and buccal mucosal cells [8].

This selective adherence is thought to contribute in part to predominance of *C. albicans* colonization and infection in human hosts [9].

Most candidal species are known to produce virulence factors including protease factors.

The ability of the various yeast forms to adhere to the underlying epithelium is an important step in the production of hyphae and tissue penetration [1].

In cases in which the epithelial barrier or host immunity is impaired, *Candida* species may cause opportunistic infections of the skin and mucosal cavities. 

Initial events of cutaneous candidiasis include adherence of blastoconidia to epithelial cell surface, fungal proliferation and colonization, and invasion of epithelial tissue [10].

In vitro studies show that adherence to corneocytes [11] and mucosal cells [12] in most cases is associated with pathogenic species.

In rodent models, it was shown that the same species are capable of hyphal invasion of corneocytes and stratum corneum to produce infection [2].

Non pathogenic species adhere poorly to corneocytes or mucosal cells [2–7] and do not invade the stratum corneum in animal models [13].

Some studies concerning the infectivity and pathogenicity of *Candida* used animals models for investigating pathogenic mechanisms [6, 7]. 

As an alternative to these models, other researchers used noninvasive methods obtaining separate corneocyte cells and used them as skin surface for *Candida* infection [5, 6, 9].

In order to characterize and compare initial adherence of *C. albicans*, *C. tropicalis,* and *C. parapsilosis*, we have used a skin model of ex vivo skin sections. We infected those skin sections with the 3 species of *Candida* mentioned above and examined them by scanning electron microscopy [14].

In the present study, we are reporting observations on the way *Candida *infect the stratum corneum in a skin model, which is a unique model that mimics the human skin.

## 2. Materials and Methods

### 2.1. Skin Sections

Normal thigh skin was harvested from skin surgically excised from women that underwent abdominoplasty. All specimens were prepared by putting pieces of skin, approximately 1 cm^2^, with full epidermal thickness into small (60 mm) petri dishes. The skin pieces were immersed in cold sterilized skin graft fluid (SGF) that was proved as adequate storage medium, which prolongs ex vivo skin viability for 2-3 weeks. Whenever possible, the skin was used within 1 h of its removal.

### 2.2. Skin Preservation (Storage) Medium

Skin graft fluid (SGF) at pH 6.4 was prepared using concentrated balanced salt solution 8.0 mL which is composed of NaCl 8.0 g/l, KCl 0.4 g/l, dibasic sodium phosphate 7H_2_O 0.0875 g/l, nonbasic potassium phosphate 0.0625 g/l, magnesium sulphate 7H_2_O 0.2 g/l, and dextrose anhydrous 1.0 g/l, all dissolved in H_2_O and sterilized, normal human plasma AB or A 20.0 mL and 0.5% neomycin sulphate in 72.0 mL of distilled water.

### 2.3. Skin Viability

The criteria for determining the viability of the skin were based on histological tests detailed by Peled et al. [15]. The extent of epidermal/dermal attachment at the interface line was observed.

### 2.4. Skin Inoculation

Fifty microliter of each fungal strain were spread equally over the skin surface (stratum corneum). The samples were incubated for 1 day to 5 days, at 30°C. The storage medium (SGF) was changed daily. Two different concentrations of blastoconidia,10^4^ mL^−1^(low) and 10^6^ mL^−1^ (high) in triplicates were used. The inoculum was prepared from a plate containing Sabouraud medium and incubated for 48 hours. Thereafter the proper concentrations were estimated by viable counting.

Controls: skin sections without *Candida* were incubated under similar conditions.

### 2.5. Electron Microscopy

Scanning electron microscopy was used for examining the inoculated skin specimens.

**Scanning Electron Microscopy (SEM)**

specimens were fixed overnight in 2.5% glutaraldehyde in 0.1 M sodium cacodylate buffer (pH 7.2) at 4°C, rinsed three times with PBS; and postfixed with 2% osmium tetraoxide for 2 h. Samples were then dehydrated in graded concentrations of ethanol (25–100%) and air-dried. The samples were viewed on an HR-SEM, ultraplus, Carl Zeiss SMT, Gemini.

### 2.6. Cultures

Clinical isolates of *Candida albicans*, *Candida tropicalis,* and *Candida parapsilosis* were obtained from Microbiology Laboratory of Rambam Health Care Campus and identified by sugar assimilation and fermentation criteria. They were maintained on Sabouraud dextrose agar slants containing 0.05 mg/mL chloramphenicol.

## 3. Results

### 3.1. *Candida albicans*

**Figure 1:**
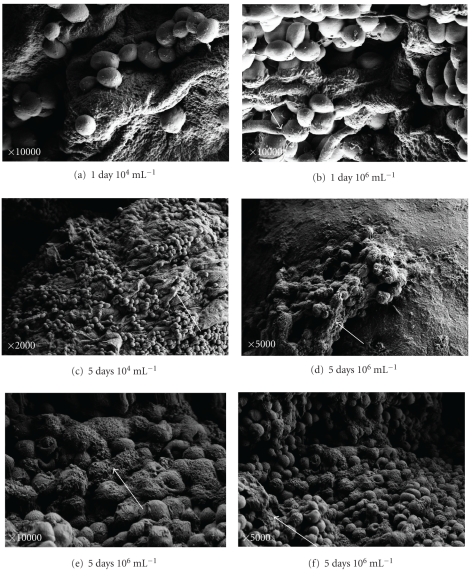
SEM of *Candida albicans. *Scanning electron micrographs (SEM) of *Candida albicans* after 1 day at 10^4^ mL^−1^ (a) and 10^6^ mL^−1^ concentrations (b) and after 5 days of incubation at 10^4^ mL^−1^ (c) and at 10^6^ mL^−1^ concentration of blastospors ((d), (e), and (f)). Large amounts of *C. albicans* are connected to the damaged skin. After 5 days the yeasts were covered probably by biofilm.

*C. albicans* adhered to human epidermis in greater numbers than the other species at every time point studied (after 24 hours and after 5 days) and with both concentrations (10^4^ mL^−1^ and 10^6^ mL^−1^). Large amount of *C. albicans* proliferated on the skin, already after 1 day at low concentration (Figure 1(a)). A higher number of yeasts appeared in higher concentration (Figure 1(b)). After 1 day and to a greater extent after 5 days, most of the *C. albicans* blastoconidia were coated with amorphous material linking adjacent blastoconidia and blastoconidia with the corneocyte surface.

The material had a strand-like appearance (in past studies “cohesion”) (Figures 1(a) and 1(b)).

Pseudohyphae elements are observed after 1 day and after 5 days (Figures 1(b) and 1(c)).

After 5 days, huge amounts of blastoconidia are shown, covering almost the entire skin model, and the blastoconidia are embedded in a layer that resembles biofilm (Figures 1(d), 1(e), and 1(f)).

### 3.2. *Candida tropicalis*

**Figure 2:**
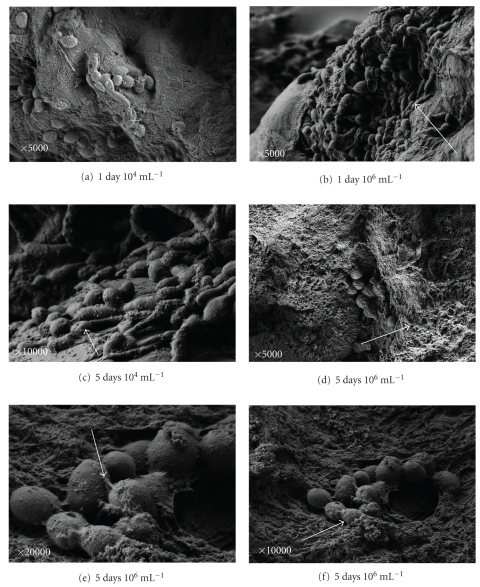
SEM of *Candida tropicalis. *SEM of *Candida tropicalis* after 1 day at 10^4^ mL^−1^ (a) and 10^6^ mL^−1^ (b). After 5 days at 10^4^ mL^−1^ (c) and 10^6^ mL^−1^ ((d), (e), and (f)) the skin is damaged and cells are adhered and “integrated” into the skin.

Low amounts of blastoconidia are observed with *C. tropicalis* in comparison to *C. albicans* (Figure 2(a)).

After 5 days at the higher concentrations the blastoconidia were coated with the material linking adjacent blastoconidia (Figures 2(e) and 2(f)), and they were covered probably with a biofilm (Figure 2(d)).

Pseudohyphae are observed after 1 day at the higher concentration and also after 5 days (Figures 2(b) and 2(c))

### 3.3. *Candida parapsilosis*

**Figure 3:**
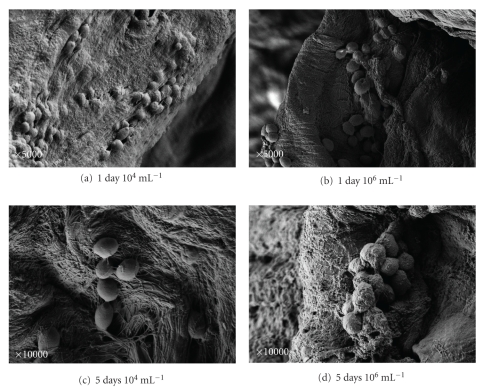
SEM of *Candida parapsilosis. *SEM of *Candida parapsilosis* after 1 day incubation at 10^4^ mL^−1^ (a) and 10^6^ mL^−1^ (b) and after 5 days at 10^4^ mL^−1^ (c) and at 10^6^ mL^−1^ (d). Although the adhesion is well defined, only small amounts of yeasts are observed. The skin is damaged and the cells are embedded by thread-like appendices.

After 1 day at low and high concentrations, only few blastoconidia were observed (Figures 3(a) and 3(b)).

After 5 days of incubation, more blastoconidia adhered in both concentrations, some kind of “threads” are shown, and the cells are embedded in the surface layer (Figures 3(c) and 3(d)).

After 1 day we were able to see characteristics that resemble minimal biofilm formation in all *Candida* species with no difference in both concentrations; this biofilm layer increased significantly after 5 days in all the species (Figures 1(c), 1(d), 2(d), 2(e), 3(c), and 3(d)).

### 3.4. Control Skin

**Figure 4:**
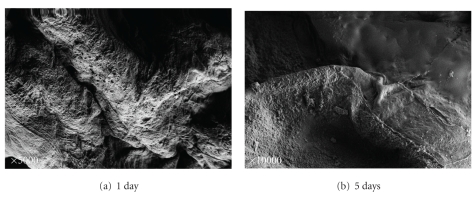
SEM of control skin. SEM of normal skin after 1 day and 5 days ((a), (b)). The skin without treatment served as control. The skin is well preserved.

After 1 day and after 5 days we observed normal epidermis (Figures 4(a) and 4(b)).

## 4. Discussion

In this study on the human skin ex vivo model, we demonstrated that* C. albicans* exhibited marked adherence to the skin in comparison to *C. parapsilosis* and *C. tropicalis* at two time points and in different concentrations.

Adhesion is provided partially by mucopolysaccharide molecules and can be evaluated qualitatively by microscopic examination of interactions between the yeast and the epithelium or quantitatively by measuring the specific adhesion of *Candida* to cultured epithelial cells [16, 17].

*C. albicans* selectively adheres to buccal and vaginal epithelial cells in humans, and adherence may play a critical role in the pathogenesis of mucocutaneous candidiasis [18]. 

In experimental rodent cutaneous candidiasis using *C. albicans*,* C. stellatoidea*, *C. tropicalis*, *C. parapsilosis,* and *C. krusei*, the only pathogenic species after topical application to intact epidermis were *C. albicans* and *C. stellatoidea* [16]; *C. tropicalis*, *C. parapsilosis,* and *C. krusei* exhibited minor or negligible adherence to epidermal corneocytes and were nonpathogenic to skin. Those *Candida* species are occasionally systemic pathogens associated with septicemia [16, 18].

In concordance with past studies, the present study ultrastructural visualization of adherence demonstrated amorphous material coating blastoconidia, mainly of *Candida albicans *and minimally of *C. parapsilosis* and *C. tropicalis*.

In those past studies this material was called cohesin. This cohesin was found as a film of loosely adherent strandlike material on the corneocyte surface of newborn mouse skin, in association with adherent blastoconidia, and was seen only with *C. albicans* and *C. stellatoidea* and not with *C. parapsilosis* and *C. tropicalis* [10].

 In our study, all *Candida *species have shown characteristics resembling biofilm formation. There is evidence that the majority of *Candida* species (*albicans* and non-*albicans*), have the capacity of producing significant amounts of biofilm and this capacity may reflect the pathogenic potential of the isolates [19].
